# Learning to tango with four (or more): the molecular basis of adaptation to polyploid meiosis

**DOI:** 10.1007/s00497-022-00448-1

**Published:** 2022-09-23

**Authors:** Kirsten Bomblies

**Affiliations:** Plant Evolutionary Genetics, Institute of Plant Molecular Biology, Department of Biology, ETH Zürich, Zurich, Switzerland

**Keywords:** Allopolyploid, Autopolyploid, Adaptation, Meiosis, Polyploidy

## Abstract

Polyploidy, which arises from genome duplication, has occurred throughout the history of eukaryotes, though it is especially common in plants. The resulting increased size, heterozygosity, and complexity of the genome can be an evolutionary opportunity, facilitating diversification, adaptation and the evolution of functional novelty. On the other hand, when they first arise, polyploids face a number of challenges, one of the biggest being the meiotic pairing, recombination and segregation of the suddenly more than two copies of each chromosome, which can limit their fertility. Both for developing polyploidy as a crop improvement tool (which holds great promise due to the high and lasting multi-stress resilience of polyploids), as well as for our basic understanding of meiosis and plant evolution, we need to know both the specific nature of the challenges polyploids face, as well as how they can be overcome in evolution. In recent years there has been a dramatic uptick in our understanding of the molecular basis of polyploid adaptations to meiotic challenges, and that is the focus of this review.

## Introduction

Whole genome duplications have been common in the history of eukaryotes and are thought to contribute to evolutionary novelty, genome complexity and adaptation (e.g. see Otto and Whitton 2000; Soltis et al. 2003; Comai 2005; Flagel and Wendel 2009; Parisod et al. 2010; Arrigo and Barker 2012; Van de Peer et al. 2017). Polyploidy has occurred in every eukaryotic kingdom, but is particularly rampant in plants (Ramsey and Schemske 1998; Soltis et al. 2003; Mable 2004; Van de Peer et al. 2017; Roman-Palacios et al. 2021), though the likelihood that any given polyploid lineage will survive is apparently quite low (Arrigo and Barker 2012). Many of our most important crops are also polyploid, which may have been under selection in some species, as polyploidy can contribute to heterosis, larger fruit or grain size, and/or greater stress resilience (e.g.Comai 2005; Udall and Wendel 2006; Renny-Byfield and Wendel 2014; Bomblies 2020); indeed, recent evidence suggests that polyploids are over-represented among crop species (Salman-Minkov et al. 2016).

A puzzling feature of polyploids is that despite their evolutionary prevalence, when they first form they face substantial challenges. Genome duplication increases DNA content as well as increased nuclear and sometimes cell volume, which can have profound effects on organismal physiology (Ramsey and Schemske 2002; Doyle and Coate 2019; Bomblies 2020). In addition, the increase in chromosome number can cause chromosome pairing, recombination and segregation problems in meiosis, which can lead to decreased fertility (Comai 2005; Bomblies et al. 2015, 2016). Some of the changes associated with genome duplication can be evolutionary opportunities (like high heterozygosity, genetic redundancy, or the increased stress resilience that often accompanies genome duplication), while others (like the issues faced in meiosis or physiology) represent challenges that must be overcome (Comai 2005; Bomblies et al. 2015, 2016; Van Drunen and Husband 2018; Bomblies 2020). There has been exciting progress recently in identifying genes and molecular functions that improve polyploid meiotic stability. These insights can provide fundamental insights into chromosome behavior and genome maintenance, and can also help open a path for employing polyploidy as a novel tool in future crop improvement by improving neopolyploid fertility (Udall and Wendel 2006; Maherali et al. 2009; Doyle and Coate 2019; Bomblies 2020).

In this review, I will first describe the different kinds of polyploids, followed by a general description of the relevant features of meiosis. This will contextualize the subsequent description of the problems polyploids face in meiosis and the cytological solutions that have evolved. Then I will discuss case studies where we know something about the molecular basis of meiotic stabilization. I will end by discussing how polyploids may get past the sometimes-devastating challenges of their teething phases. Much of what I discuss focuses on studies that have mostly or exclusively focused on male meiosis. This is because in plants male meiosis is much easier to analyze cytologically. However, there is also some evidence that at least in autopolyploids, male meiosis may in fact also be more sensitive to polyploid challenges (Koul and Raina 1996). Why this would be, and whether this trend holds true for other polyploids remains, to my knowledge, unexplored.

## Defining the two major types—auto and allopolyploids

**Fig. 1:**
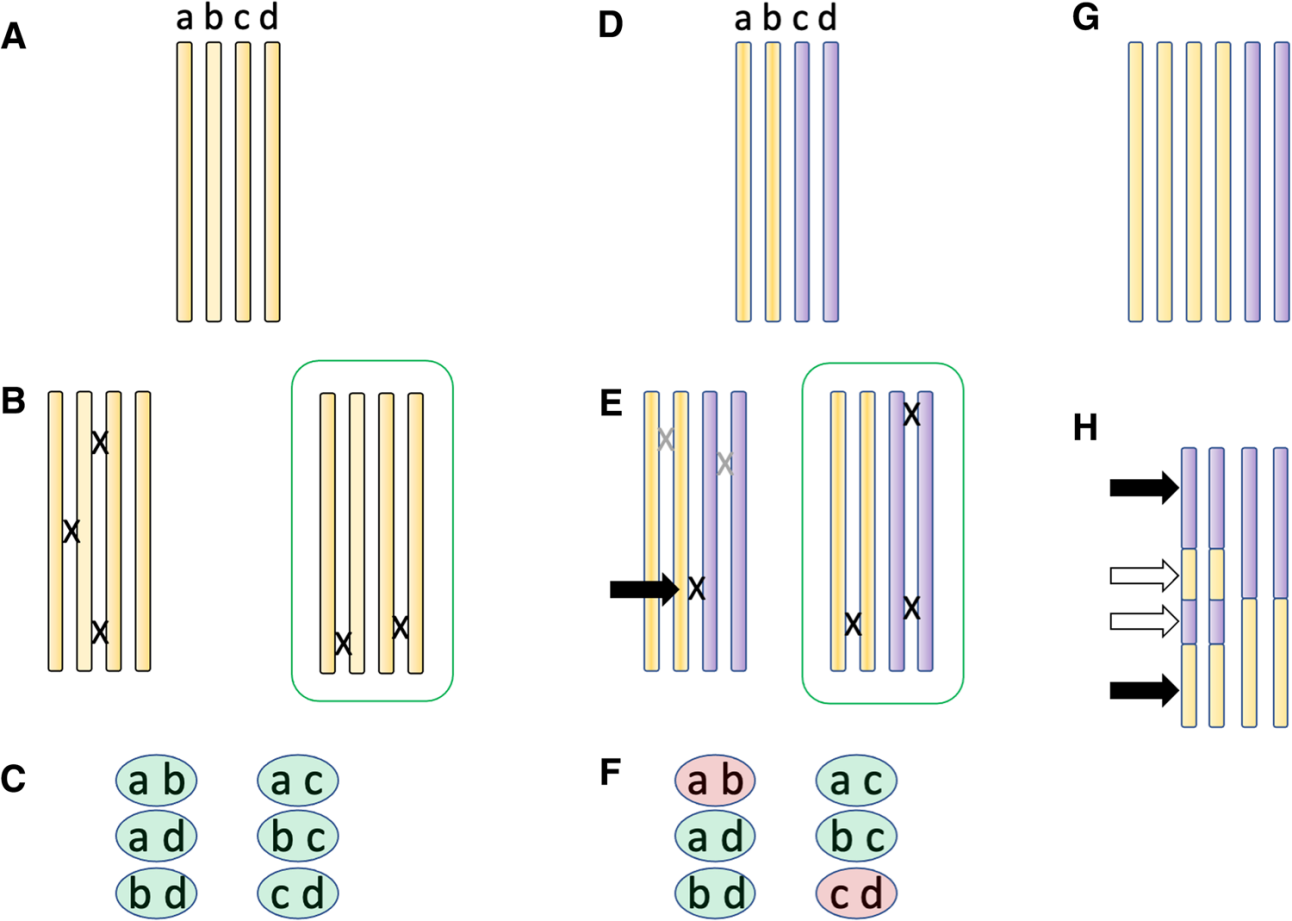
Different kinds of polyploids, and their major challenges. (**A**) Autopolyploids contain multiple (here four, an autotetraploid) equally homologous copies of each chromosome. (**B**) Problems arise when recombination among the four copies gives rise to multivalents, especially the trivalent / univalent example shown here at left. In evolved (meiotically stable) autopolyploids, crossovers tend to occur on the group of homologs such that only bivalents persist to metaphase I (configuration in box). (**C**) Possible gametes: As a result of random crossover partner choice, stable autopolyploids can make every possible combination of homologs in their gametes. (**D**) Allopolyploids have two distinct sub-genomes, such that an allotetraploid (illustrated here) will have two sets of homologs (yellow vs. purple); similar chromosome copies from different sets are called homeologs. For example, chromosome a and c are homeologs, and a and b are homologs. (**E**) Problems arise in meiosis when recombination occurs among homeologs (black “X”, black arrow), as this can lead to mosaicism in which recombination partner choice becomes inconsistent (see text). Stable allopolyploids (green box) have recombination events occurring only among homologs (here, no exchanges among any yellow and any purple chromosome). (**F**) Possible gametes: Homeologous recombination can yield gametes carrying both alleles from just one subgenome (red gametes, ab, cd), while stable disomic inheritance will yield only gametes that carry one chromosome from each subgenome (green gametes, all remaining combinations). (**G**) An example of an auto-allo-polyploid genome. The four yellow chromosomes are homologous and can behave like the situation in panels **A**–**C**, while the two purple chromosomes would behave, with respect to the yellow ones, like in panels** D**–**F**. (**H**) A segmental allopolyploid. In these situations, the chromosome is a mosaic of regions that are allopolyploid (e.g. two yellow and two purple copies) and regions that are homozygous for one or the other parent. If crossovers occur in the regions indicated by the black arrows, there may be no partner preference, and markers in that region will show tetrasomic inheritance, while regions indicated by white arrows will show disomic inheritance

Polyploids are generally defined as coming in two distinct types, auto- and allo-polyploids (Fig. 1). Either type of polyploidy can arise either from somatic genome duplication (in plants this can be heritable because plants do not sequester their germline), or by the production and fusion of unreduced gametes, the production rate of which can dramatically increase under stressful conditions (Ramsey and Schemske 1998, 2002; De Storme et al. 2012; De Storme and Geelen 2014). Nowadays auto- and allopolyploids are primarily distinguished by their origin and/or genetics (Ramsey and Schemske 1998, 2002; Bomblies and Madlung 2014). By the “origins” definition, allopolyploids have a hybrid origin and thus carry two or more distinct sets of chromosomes called “sub-genomes,” while auto-polyploids arise within species and have multiple roughly equivalent homologous copies of each chromosome.

In addition to the origin of a particular polyploid, it is important to know the chromosome segregation behavior of a polyploid for understanding the challenges it faces. Segregation can be tested by following marker segregation in experimental crosses (e.g. Jelenkovic and Hough 1970; Krebs and Hancock 1989; Rieseberg and Doyle 1989; Wolf et al. 1989), and in the age of genome resequencing, also by analyzing expected and observed genome-wide genotype frequencies under different inheritance models (e.g. see Hollister et al. 2012). By the “genetic” definition, established allopolyploids almost always have disomic segregation, meaning chromosomes recombine (and are thus linked in metaphase I) preferentially with more similar homologs from the same sub-genome. Established autopolyploids tend to have polysomic segregation (“tetrasomic” if there are four chromosome copies), meaning the chromosomes have no partner preferences for recombination and chiasma formation, yet these species often still form only bivalents in metaphase I (e.g. Jelenkovic and Hough 1970; Krebs and Hancock 1989; Rieseberg and Doyle 1989; Wolf et al. 1989; Hollister et al. 2012). As a result of their respective inheritance patterns, only allopolyploids have “duplicate genes”, while autopolyploids segregate more alleles at each (non-duplicated) locus. Because this review is about meiosis, I will generally weigh chromosome segregation more heavily than origins of a particular lineage when considering established polyploids, and origins more heavily when considering neopolyploids, as this is important for understanding the problems they face early on.

Some confusion arises in the older literature, as polyploids were often defined cytologically. Anything that formed only bivalents in metaphase was considered allopolyploid, and anything that formed even a few multivalents in metaphase as autopolyploid. Now, however, we know autopolyploids with fully tetrasomic inheritance can also form exclusively or primarily bivalents in metaphase I (e.g. Dawson 1941; Jelenkovic and Hough 1970; Krebs and Hancock 1989; Rieseberg and Doyle 1989; Wolf et al. 1989). Thus, many polyploids described in the earlier literature may be mis-classified, contributing to what might be a considerable under-counting of auto-polyploids (Soltis et al. 2007). We have since refined our classification of auto- vs. allo-polyploids as well as our understanding of their genetics. Contextualizing this information with our modern understanding of meiosis has led to a much-improved ability to characterize both problems and solutions, as well as assess their predictability. Finally, auto- and allo-polyploids are often said to have a range of intermediates between the extremes (Stebbins 1947; Chen and Ni 2006; Stift et al. 2008). I discuss in Box 1 what this may mean in the context of the meiotic adaptations described here.

## Basic progression of meiosis in diploids

To contextualize the problems that polyploids face in meiosis, I will first briefly describe the relevant parts of the general progression of events in diploid meiotic chromosome pairing, recombination and segregation. A comprehensive view of meiosis is not possible here. For deeper and broader discussions of meiosis see e.g. (Zickler and Kleckner 1999, 2015, 2016; Keeney and Neale 2006; Lynn et al. 2007; Mercier and Grelon 2008; Lam and Keeney 2014; Hunter 2015; Mercier et al. 2015).

**Fig. 2:**
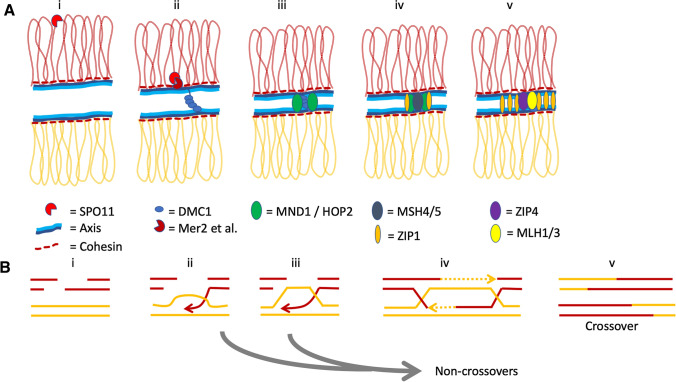
Diagram of core features of meiosis relevant to this paper. (**A**) Structural view. (**B**) Diagramatic view. Homologs in red and yellow. After chromosomes are replicated to give identical sister chromatids (i) the chromatids are linked by cohesin complexes and organized in chromatin loops. The cohesin complexes containing the meiosis-specific subunit REC8 in turn also recruit the structural axis components (e.g. Red1, Hop1 in yeast, and ASY1, ASY3 and ASY4 in *Arabidopsis*). Double strand breaks are created by SPO11 (ii), and together with Mer2 and other proteins, brought to the chromosome axes while ends are resected and processed into single strand filaments that are coated with Rad51 and the meiosis-specific DMC1 (ii). Mediated by DMC1, single strands invade other chromosomes and “search” for homologous sequences. This search and subsequent stabilization are mediated by the MND1/HOP2 complex (iii). Strand invasions are then processed by ZMM proteins, including MSH4 and MSH5 (which are recruited by ZIP1) (iv), and ZIP4 and others into Holliday Junctions and later crossover events (iv-v). Grey arrow: The vast majority of pre-recombination events are shunted to non-crossover fates at various stages

The key events of meiosis relevant to the discussion of polyploid adaptations occur early in meiosis I, and result in chromosome co-alignment (pairing) and the initiation and maturation of homologous recombination events (Fig. 2). This process begins after chromosomes are replicated to yield two identical sister chromatids. Sister chromatids are held together by, among other things, cohesin complexes that contain a meiosis-specific subunit, REC8 (Molnar et al. 1995; Michaelis et al. 1997; Watanabe and Nurse 1999). REC8-containing cohesin recruits chromosome axis proteins (Molnar et al. 1995; Sakuno and Hiraoka 2022). The axes are a linear proteinaceous structure conserved across eukaryotes that extend the entire length of each set of paired sister chromatids in meiosis. The axes form a context on which the earliest events of recombination play out, and help direct the choice of the homolog for recombinational interactions instead of the sister chromatid (Schwacha and Kleckner 1997; Zickler and Kleckner 1999, 2015; West et al. 2019). In many species, axis length correlates positively with recombination rate (Kleckner et al. 2003; Ruiz-Herrera et al. 2017; Song et al. 2021).

One of the first events in recombination itself is programmed formation of hundreds of double strand breaks along the chromosomes by SPO11 (Keeney and Neale 2006; Lam and Keeney 2014). Double strand breaks are then processed such that long single-stranded DNA “tails” are created, which become coated by Rad51 and Dmc1 (only DMC1 is meiosis-specific) (Fig. 2). These proteins help guide a process called single end (or strand) invasion, in which the coated single-stranded DNAs “invade” other chromosomes and identify regions of homology. The MND1/Hop2 complex then interacts with DMC1 to stabilize single-strand invasions, and also to reject events where heterozygosity is too high, thus preventing ectopic recombination (Gerton and DeRisi 2002; Tsubouchi and Roeder 2002; Kerzendorfer et al. 2006; Panoli et al. 2006; Pezza et al. 2007; Vignard et al. 2007; Zhao et al. 2013). Once several strand invasion events stabilize along a chromosome, the chromosomes are co-aligned (“paired”). The MSH4/MSH5 complex also associates early with pre-recombination interactions and helps stabilize them (e.g.Ross-Macdonald and Roeder 1994; Novak et al. 2001; Argueso et al. 2004; Higgins et al. 2004; Shinohara et al. 2008). The recruitment of MSH4/MSH5 to recombination interactions requires ZIP1, a member of the so-called ZMM groups of proteins (Borner et al. 2004; Lynn et al. 2007; Higgins et al. 2008; Voelkel-Meiman et al. 2015). As meiosis progresses, additional ZMM proteins associate with the pre-recombination interactions and regulate their fate as either crossover or non-crossover events (Lynn et al. 2007; Shinohara et al. 2008). ZMM proteins are only required for the so-called Class I crossovers, which are the majority in most species. These crossovers (unlike Class II crossovers) generate and are sensitive to crossover interference, which prevents crossovers forming near one another (Copenhaver et al. 2002; Zickler and Kleckner 2016).

Box 1: Intermediates between auto- and allopolyploid extremes Since Stebbins put forward the idea in 1947 (Stebbins 1947), the statement is often made that there is a continuum of intermediates between auto- and allopolyploids. Sybenga argued nearly 50 years later, that such intermediates are in fact rare, and the vast majority of established polyploids are either solidly auto- or allopolyploid (Sybenga 1996). What is the evidence now? In terms of polyploid origin, every intermediate of genetic divergence between “different species” and “within species” is of course possible. But in terms of chromosome segregation behavior and meiotic adaptations, probably not. In genetic terms, there are three discrete types of “intermediate” that in principle could exist: (1) auto-allo-polyploids where auto- and allo-polyploid sub-genomes co-exist and remain distinct at the chromosome level, (2) segmental allopolyploids where some chromosome regions preferentially pair/recombine while others do not, or (3) recombination partner choice could be partly preferential.

*1. Auto-allo-polyploids*. There are examples of species with complex genomes where autopolyploid genomes co-exist with more diverged sub-genomes that they do not recombine with (Fig. 1G), e.g. a genome constitution such as “AAAA/BB” where the A chromosomes can recombine freely with each other as in an autotetraploid, but do not recombine with the B chromosomes, which in turn also only recombine amongst themselves. Examples include the grasses sugar cane, *Pennisetum squalidum*, and *Festuca kingii* (e.g. Boyle 1950; Patil et al. 1961; Premachandran et al. 2011; Bock et al. 2014). Auto-allo-polyploids can arise for example from hybridization between autotetraploids and either a diploid or an allopolyploid, followed by genome doubling. To date we know little about what adaptations it takes for this sort of polyploid to stably undergo meiosis; it may well require the co-existence of both auto- and allo-polyploid adaptations.

*2. Segmental allopolyploids.* In “segmental allopolyploids” the genome is a mosaic of regions that are allopolyploid and autopolyploid in their recombination and segregation behavior (Fig. 1H). Such a situation can arise in an allopolyploid when homeologous recombination homozygoses some parts of the genome (e.g. Leal-Bertioli et al. 2018; Mason and Wendel 2020). Can different regions of the same chromosome show different patterns of inheritance? It seems the answer is sometimes yes. One example is in the genome of polyploid trout, where most of the genome shows disomic inheritance, but one terminal region is tetrasomic (Allendorf and Danzmann 1997), showing recombination among all four chromosome copies can occur in this region. Intermediacy is thus only at the genome-wide level—any given region is discretely auto- or allopolyploid in its genetic behavior. How stable this situation is, or what adaptations it requires, remains mostly unknown.

*3. Intermediate or partial pairing/recombination partner preference*. In theory, one can imagine that individuals have some, but not absolute, preferences for recombining particular chromosomes (Stift et al. 2008; Hollister et al. 2012; Meirmans and Van Tienderen 2013). This would lead to genome- or at least chromosome-wide “intermediate” inheritance between the disomic and tetrasomic extremes. While appealing as a model, it is, however, likely to be at most transient, since even small amounts of non-preferential recombination can rapidly homogenize the genome (Muramatsu 1990; Sybenga 1996; Meirmans and Van Tienderen 2013), generating chimeric chromosomes whose recombination partner choice will depend on where recombination events are located along the chromosome. Thus, it is perhaps unsurprising that the only known examples of this kind of intermediate-preference system are recent hybrids, including interspecies hybrids in *Rorippa*, sugar cane and *Acacia* (Jannoo et al. 2004; Stift et al. 2008; Xie et al. 2015; Le et al. 2021). However, even if they are likely evolutionarily ephemeral, such systems can teach us interesting things about the effects of hybridization in polyploid systems, and how higher ploidy levels and/or complex auto-allopolyploids can evolve. It seems unlikely that there are adaptations that could stabilize this sort of intermediate system in the long term.

## Allopolyploids

### The problem(s) and the cytological solution(s)

Allopolyploids start life already with distinct “sub-genomes” from the two parents (Fig. 1). The more similar chromosome copies within a sub-genome are called “homologs” (as in diploids), while the less similar copies from distinct sub-genomes are called “homeologs”. In most established allopolyploids, sub-genomes remain genetically distinct, because in metaphase I, bivalents consist of the more similar homologs, and these then segregate from each other (disomic inheritance), leading to permanent heterozygosity for the two sub-genomes (Pikaard 2001; Bomblies and Madlung 2014). But this is not necessarily the case from the beginning. We will see below, there is clear evidence from mapping crosses among stable allopolyploids and neo-allopolyploids that preferential recombination within subgenomes has a genetic basis and is a derived rather than an innate feature of meiosis.

Homeologous recombination (recombination among sub-genomes) can cause multiple problems. It can yield meiotic multivalents, and after segregation, gene loss, mosaic genome homogenization, and aneuploidy (Feldman and Levy 2009; Szadkowski et al. 2010; Zhang et al. 2013; Gou et al. 2018; Wu et al. 2020). For example, in wheat, neo-allo-hexaploids acquire frequent whole-chromosome aneuploidies, and occasional “cryptic aneuploidies” where one chromosome was lost and another gained (Zhang et al. 2013). Interestingly, one of the sub-genomes is more stable, perhaps because it is less likely to undergo homeologous recombination with the other two. Neo-polyploid (resynthesized) *Brassica napus*, undergoes so much restructuring and homeologous exchange, that it has been described as a “genome blender” (Song et al. 1995; Szadkowski et al. 2010; Gaebelein et al. 2019). There may be other problems as well, for example, in wheat, when multivalents persist to metaphase, there are also lots of unresolved interlocks, suggesting there might be some relationship between the processes that eliminate both types of structures (Hobolth 1981; Holm and Wang 1988), while in *A. suecica* there are subtle, but persistent instabilities unrelated to homeologous recombination (Nibau et al. 2022).

Some of the neo-allopolyploid instabilities described above are so extreme that it can become difficult to reconcile, either with the absence of evidence for bursts of extensive homeologous exchange in the history of extant allopolyploids, or with the long-term maintenance of distinct sub-genomes. For example, both natural allopolyploid cotton and *Arabidopsis suecica* maintain separate subgenomes. Though both species do continue to accumulate rearrangements from homoeologous exchanges at a slow pace, it seems neither had a dramatic burst of instability immediately after polyploidy (Salmon et al. 2010; Burns et al. 2021). How do we explain the dramatic events we see in lab-generated neo-polyploids in the context of what seems to be relative stability of natural ones even from their beginnings? A clue comes from *Brassica napus*, where it was found that the extent to which the genome rearranges in synthetic neo-allopolyploids varies substantially depending on the diploid genotypes used, suggesting there are genetic variants segregating in the diploid progenitors that affect meiotic stability of polyploids derived from them (Attia and Röbbelen 1986; Szadkowski et al. 2010). Perhaps the allopolyploid lineages that survive are those that are from the start less inclined to genome rearrangement. Whether stabilizing alleles are selected from standing variation or de novo during allopolyploid evolution, there is nevertheless clear evidence that there is a genetic basis to the preference for recombining with homologs over homeologs.

### The molecular solution(s) to allopolyploid meiosis

In most allopolyploid species, the decision of which chromosomes will recombine seems to occur after the point at which chromosomes are paired and co-aligned. The evidence for this is that in many allopolyploids, homologs and homeologs co-align and even form multivalent associations in pachytene (e.g. Hobolth 1981; Loidl 1988; Martinez et al. 1996), but recombination events then only mature on the more closely related homologs. How is this achieved? I turn now to a few example species to examine what is known about the genetic and molecular basis of allopolyploid stabilization.

*Triticum aestivum* (bread wheat): Bread wheat is an allohexaploid that arose from a merger of three distinct genomes, has disomic inheritance, and bivalents in metaphase I that are comprised of only homologs, not homeologs (Riley and Chapman 1958; Sears 1976; Juahar et al. 1991). Nevertheless, during the first stages of meiosis (in prophase I), wheat can form multiple-chromosome alignments and multivalents (Hobolth 1981; Martinez et al. 2001), suggesting that initial chromosome “pairing” is actually indiscriminate, and the decision point comes later (during crossover maturation). In contrast to established wheat allohexaploids, newly synthesized allo-polyploids instead have rampant homeologous recombination, showing that preferential recombination partner choice is an evolved feature (Zhang et al. 2013). Multiple loci have been identified that contribute to preferential recombination in wheat (Riley and Chapman 1958; Sears 1976; Martinez et al. 2001; Koo et al. 2017), and now, happily, two of the causal genes have been identified, which helps refine models of how preferential chromosome recombination and segregation is achieved in wheat.

The strongest of several genetic loci that drives preferential recombination of homologs over homeologs is *Pairing homologous 1* (*Ph1*; Riley and Chapman 1958; Luo et al. 1996). (See Box 2 for a description why preferential “pairing” is probably no longer an accurate description in most species). Non-*Ph1*-containing lines recombine homeologs as well as homologs, which results in multivalent formation, chromosome mis-segregation, and deleterious homeologous exchanges (Holm and Wang 1988). While the genetic behavior of *Ph1* has been studied for decades, the mystery of its molecular identity was only recently solved: the *Ph1* region is large and complex, but a (or the) causal gene seems to be a diverged extra copy of a gene encoding a ZMM group protein called ZIP4 (Rey et al. 2017; Martín et al. 2021). Like other ZMM proteins, ZIP4 is essential for Class I interfering crossover formation and defines crossover fate decisions early in pre-recombination maturation (Tsubouchi et al. 2006; Lynn et al. 2007; Shinohara et al. 2008; Shen et al. 2012). Thus, the ZIP4 allele encoded by *Ph1* in wheat could have evolved greater sensitivity to polymorphism in regulating the decision whether to progress a pre-recombination interaction to a crossover or non-crossover fate. How exactly the diverged ZIP4 protein encoded by the duplicated gene at the *Ph1* locus might have evolved an altered sensitivity to polymorphism will be exciting to test.

A second locus that helps prevent recombination among homeologs is *Ph2*, and the likely causal gene has recently been shown to be MSH7 (Serra et al. 2021). MSH7 is a plant-specific mismatch recognition protein active in meiosis that forms a complex with MSH2, and likely arose via an ancient duplication of the eukaryote-wide MSH6 gene early in plant evolution (Culligan and Hays 2000; Culligan et al. 2000). Silencing the *Ph2* copy of MSH7 in wheat/*Aegilops* hybrids, whose chromosomes do not normally recombine, resulted in at least a five-fold increase in homeologous recombination (Serra et al. 2021). This result suggests that the normal function of the *Ph2* MSH7 allele is to monitor and reject nascent recombination events with excessive mismatches. Similarly, in a tomato substitution line carrying a homeologous chromosome from a related species, reducing MSH7 activity also increases homeologous recombination (Tam et al. 2011). Like ZIP4, MSH7 coordinates recombination fate decisions and can apparently quantitatively control sensitivity to polymorphism to help discriminate homologs from homeologs during crossover maturation.

*Brassica napus (oilseed rape)*: *Brassica napus* is a young allopolyploid generated multiple times during domestication from hybridization between two diploid *Brassica* species (U 1935); it seems to be purely a product of domestication, since wild *B. napus* populations are not known to exist. As in wheat, genetic loci suppress homeologous recombination in meiotically stable established allopolyploid *B. napus* strains (e.g.Jenczewski et al. 2003; Liu et al. 2006; Cifuentes et al. 2010; Grandont et al. 2014; Higgins et al. 2021; Xiong et al. 2021). The underlying genes have not yet been identified, but some useful information about recombination and segregation stabilization is nevertheless available for this species.

As noted above, in synthetic (newly generated) allopolyploid *Brassica napus*, where any stabilizing genes would come from segregating variation from the diploid parents, seven genomic regions were associated with quantitative effects on fertility and meiotic stability, five of which contain meiosis genes as candidates (Gaebelein et al. 2019). Of 14 candidate meiosis genes, 10 contain amino acid polymorphisms differentiating diploid donor alleles. Though none have been tested functionally, the list includes intriguing candidates including the single-strand DNA-binding protein RAD51, cohesin components SCC2 and SMC1, and the mismatch repair protein MSH2 among others (Gaebelein et al. 2019). Another study also identified candidate genes underlying several stabilizing QTL, including MSH3 and a number of other meiosis genes (Higgins et al. 2021). These studies, which show that segregating genetic variants in diploids can contribute to polyploid meiotic stability, could help explain how early polyploid lineages can achieve enough stability to survive long enough to evolve additional solutions.

While we cannot make too much of unconfirmed candidates, they nevertheless allow for some speculation as to what might be possible. A particularly interesting candidate from the list is the gene encoding MSH2, which interacts with both MSH6 and MSH7 (Wu et al. 2003). In tomato, silencing of MSH2 (like MSH7) increased homeologous recombination rates (Tam et al. 2011). Consistent with a potential role in the selectivity of allopolyploid recombination partners, in *Arabidopsis thaliana* MSH2 plays a role in mediating the sensitivity of homologous recombination to polymorphism (Emmanuel et al. 2006; Li et al. 2006). In *A. thaliana* crosses, MSH2 regulates recombination such that it occurs preferentially in more polymorphic regions, but only to a point (Ziolkowski et al. 2015; Blackwell et al. 2020). That is, MSH2 seems to ensure that recombination events preferentially occur in regions that have some, but not excessive polymorphism, supporting the hypothesis that its sensitivity to polymorphism could in principle be toggled up or down in allopolyploids to allow discrimination of homologs from homeologs.

It was also recently shown that artificially reducing (but not eliminating) expression of the ZMM-group gene MSH4 in newly polyploid *B. napus* greatly reduced the rate of homeologous recombination, while not substantially affecting homologous recombination (Gonzalo et al. 2019). Like other ZMM proteins, MSH4 is required for the formation of class I (interfering), crossovers, but seems to act earlier than other ZMM proteins (e.g. Ross-Macdonald and Roeder 1994; Zalevsky et al. 1999; Novak et al. 2001; Argueso et al. 2004; Higgins et al. 2004, 2008; Shinohara et al. 2008). MSH4 is not generally considered to affect pairing, but in yeast, while in *msh4* mutants chromosome alignment (pairing) does occur, chromosomes remain spaced about twice as far apart as in wild type (Storlazzi et al. 2010), suggesting MSH4 also contributes to the establishment or stabilization of normal pairing interactions. Whether MSH4 affects partner choice at the level of pairing or recombination regulation (or both) in allopolyploid *B. napus* is as yet unclear.

*Arabidosis suecica: A. suecica* is a naturally occurring allopolyploid formed from hybridization between diploid *A. thaliana* and either diploid or autotetraploid *A. arenosa* (O’Kane et al. 1996; Jakobsson et al. 2006; Burns et al. 2021; Nibau et al. 2022). In contrast to natural (established) *A. suecica*, neopolyploids generated in the lab are meiotically unstable (albeit to varying degrees), with extensive homeologous recombination resulting in homozygosity of chromosome regions for one parent or another, aneuploidy, and chromosome mis-segregation (Pontes et al. 2004; Henry et al. 2014). Nevertheless, as noted above, in natural *A. suecica* there is no evidence for a burst of homeologous exchange having occurred (i.e. there was no “genomic shock” resulting from polyploidy), though there is some evidence that low levels of homeologous exchange have continued at a low rate throughout its evolution (Burns et al. 2021). Recent cytological data confirms that the established natural *A. suecica* is meiotically quite stable. Occasional abnormalities in recombination, synapsis and chromosome segregation, do occur, but none were attributable to homoeologous recombination events, suggesting other instabilities persist in this allopolyploid (Nibau et al. 2022). Genetic mapping in F_2_ populations from crosses between natural and synthetic *A. suecica* identified one quantitative trait locus where the allele from the established line improved cytological stability and fertility by suppressing homeologous exchange (Henry et al. 2014). The gene responsible is as yet unknown.

*Oryza sativa* (Rice): Rice is generally diploid, but tetraploid rice lines have been derived from hybrids of two subspecies. These tetraploids have high rates of multivalent formation, homeologous exchange, and chromosome mis-segregation (Xu et al. 2014; Wu et al. 2020). However, one line is meiotically stable with bivalents in metaphase I only involving homologs, not homeologs (Xiong et al. 2019). This line was found to express particularly high levels of a gene encoding a meiotic protein, MND1, which significantly reduces univalent and trivalent rates in the naturally stable line, and when overexpressed as a transgene, it rescued a previously unstable line (Xiong et al. 2019). MND1 is an interesting gene in this context. It is conserved across eukaryotes, and together with another protein, HOP2, is required for homologous recombination in fungi, plants and animals (Gerton and DeRisi 2002; Tsubouchi and Roeder 2002; Kerzendorfer et al. 2006; Panoli et al. 2006; Pezza et al. 2007; Vignard et al. 2007; Zhao et al. 2013). Structural and in vitro binding analyses suggest that HOP2/MND1 complexes directly associate with and stabilize DMC1-mediated strand invasion (Zhao et al. 2013; Kang et al. 2015; Crickard et al. 2019). Likely relevant to an allopolyploid situation is the observation that in yeast *mnd1 / rad51* double mutants have increased ectopic recombination between non-homologous sequences resulting from an increased promiscuity of DMC1-mediated strand invasion in the absence of MND1. This result implicates MND1 in increasing the “pickiness” of DMC1-mediated pairing partner choice during strand invasion (Henry et al. 2006). Thus, loss of *mnd1* makes DMC1-mediated strand invasion less sensitive to polymorphism, while overexpressing MND1 seems to make it more sensitive, and likely this allows strand invasion interactions with homeologs to be rejected. How exactly MND1 might modulate polymorphism tolerance thresholds, and why over-expressing it changes the sensitivity to polymorphism, is as yet unclear. This work highlights that studying partner choice in polyploid systems may yield a unique opportunity to learn more about fundamental aspects of meiosis, such as how sensitivity of recombination to polymorphism can be tuned, and how polymorphism is “measured” molecularly to direct recombination fate decisions.

*Repeatability:* The allopolyploid systems described above highlight that a major adaptation in allopolyploids (in fact the only one currently known) is an improved ability to discriminate more diverged homeologs from less diverged homologs as partners for recombination. At the molecular level, the cases described above highlight that there seem to be several proteins or molecular processes via which these decisions can be regulated. The relevant genes are highly conserved across eukaryotes, showing that, as happens so often in evolution, allopolyploids are not inventing something new—they are retuning a system that is already well-poised to make molecular “decisions” of this sort. What we can now say is that allopolyploids do seem to be highly predictable in the sense that they stabilize meiosis by making recombination partner choice more sensitive to polymorphism, but there are different genes they may modify to achieve this.

*Sequence divergence as an innate cue for preferential recombination (or not):* Does a recombination partner preference for homologs over homeologs imply that in established allopolyploids meiosis chromosomes will *always* “pick” more similar chromosomes as recombination or pairing partners, or is there a threshold below which anything goes? There have been some intriguing “competition” experiments designed to assess recombination partner choice in polyploid contexts, and they tell a complex story. One series of studies in tetraploid rye used chromosomes with heterochromatic “C-bands” as visible cytological markers to test whether chromosomes prefer to partner with identical or similar chromosomes when brought together in hybrids. The outcome depended on where the marker is located: A tetraploid heterozygote (AABB) for a telomeric C-Band region shows either random association or a preference for identical over homologous chromosomes, while individuals heterozygous for chromosomes with a centromere-proximal C band show preferential recombination of homologs over identical partners (Santos et al. 1983; Benavente and Orellana 1989, 1991). In another set of studies, hybrids with more divergent parents showed a tendency to prefer identical over homeologous partners, but not always (Benavente and Orellana 1991; Benavente and Sybenga 2004). In *A. thaliana* neotetraploids, a similar experiment showed there can be differences among chromosomes within an individual: In inter-accession hybrid neo-polyploids, one chromosome had no partner preference at all, while another had a preference for partnering homologous over identical chromosomes (Parra-Nunez et al. 2018). In maize, genetic crosses between two neo-tetraploid lines created from genome duplication of two different strains that were triploid for one chromosome (AAB) were used to test for recombination partner preferences (Braz et al. 2021). Though the chromosomes from the two strains can recombine in a diploid context, suggesting they have not exceeded some differentiation tolerance threshold, there is a tendency for the chromosomes from the same genetic background to recombine preferentially (Braz et al. 2021). The authors conclude from this that there is an innate tendency for a chromosome to associate with a more similar partner even when a genetic locus that suppresses homeologous pairing is lacking. However, maize has a polyploid history, so it is also conceivable that it previously adapted to polyploid meiosis, and that it retained at least some of that evolved choosiness. A very interesting point from this study, is that since the chromosomes from these two strains can in principle recombine, it seems to be the *difference* in divergence among the different available partners, and not a specific hard threshold, that is somehow being recognized to determine recombination partner choice.

Taken in aggregate, the above results suggest that variation in levels of polymorphism along chromosomes can dictate partner preferences, and also that homologous recombination may prefer some intermediate level of polymorphism, such that both too much, or too little polymorphism can inhibit progression to crossover formation. Whether there are generally set thresholds in allopolyploids, or chromosomes are somehow “assessed and compared” is not clear. Understanding how the sensitivity of partner choice is tuned to new sensitivities in allopolyploids will provide interesting new insights into how exactly DNA sequence polymorphism is measured and responded to during recombination partner choice.

Box 2: Preferential pairing versus preferential recombination The phrase “pairing preference” is common in the allopolyploid literature, but when the phrase was first coined, “pairing” was considered synonymous to associations observed in metaphase I (Alabdullah et al. 2021). Now “pairing” refers to the initial recognition and co-alignment of homologous chromosomes, which occurs much earlier (Zickler and Kleckner 2015, 2016). As a result, nowadays, the implication of the continued use of the phrase “pairing preference,” is that it is the acceptance or rejection of strand invasion that mediates homolog-specificity in allopolyploids. This may be the case sometimes, but in many systems it is either not known, or the available evidence suggests otherwise. In many allopolyploid species with stable disomic inheritance, pre-synaptic alignment of all homologs and homeologs is observed (e.g. Hobolth 1981; Loidl 1986; Rasmussen 1987; Davies et al. 1990; Jones and Vincent 1994; Khazanehdari et al. 1995; Stack and Roelofs 1996), suggesting that initial pairing interactions occur indiscriminately, at least in these systems. Afterwards, nascent pre-recombination interactions only mature into crossovers on more similar bivalents, suggesting the decision point actually comes during crossover maturation (e.g. Hobolth 1981; Loidl 1986; Rasmussen 1987; Davies et al. 1990; Jones and Vincent 1994; Khazanehdari et al. 1995; Stack and Roelofs 1996), when pairing is already firmly established (Bishop and Zickler 2004). This conclusion is further supported by the genes identified as important for preventing homeologous recombination, most of which function in some aspect of recombination maturation, though the rice MND1 story may be an example of true “preferential pairing”. In any case, it cannot be a priori assumed that it is pairing per se that is affected without further molecular analysis. I would therefore advocate using the phrase “pairing preference” only in those cases where it has been specifically molecularly demonstrated. As has also been suggested elsewhere (Parra-Nunez et al. 2018), I will use phrases like “recombination partner choice,” intending this to refer to the final outcome in metaphase I and agnostic as to the actual stage in the process of pairing or recombination maturation that is affected.

## Autopolyploid problems and solutions to meiosis

**Fig. 3:**
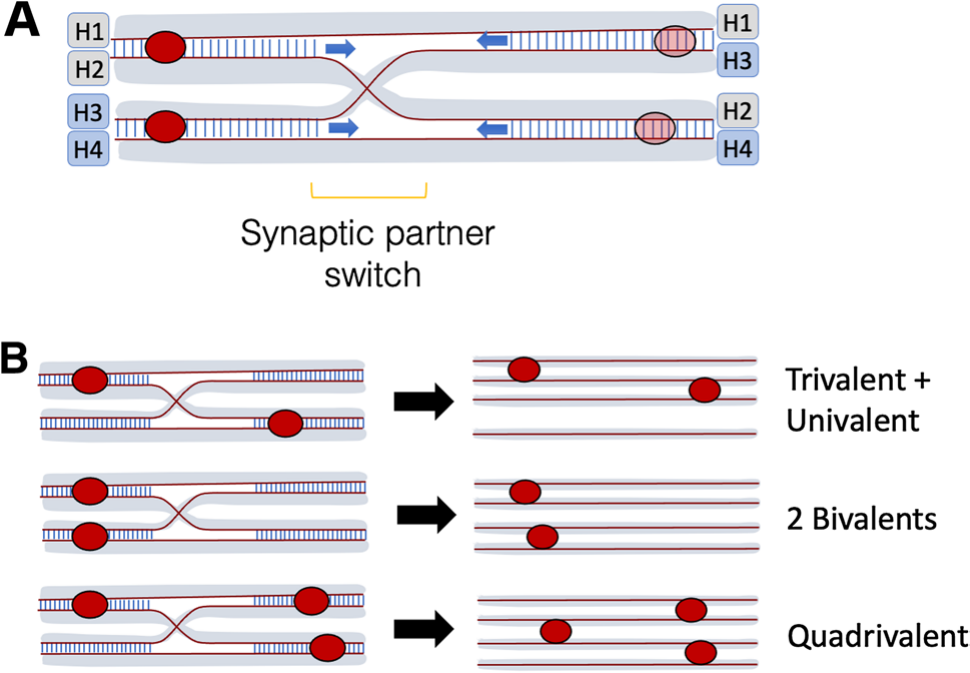
Structure of pachytene quadrivalents in polyploids and outcomes of crossover positioning. (**A**) Overview of structure of pachytene, or synaptic, multivalents commonly observed in polyploid meiosis. These arise when pairing and synapsis start at or near chromosome ends and progress inwards. When the chromosome ends pair, or begin the process of recombination, with different partners (designated H1-H4) at different sites, this forces a synaptic partner switch (SPS) in between. These sites are often accompanied by surrounding regions of asynapsis. Synapsis can either initiate from developing crossovers (solid red circles at left) or from synaptic initiation sites that do not develop true crossover events (transparent circles at right). (**B**) Crossover distribution on pachytene mutivalents dictates their metaphase I outcome, because in late pachytene, when the synaptonemal complex is removed, only crossover events hold chromosomes together. Top panel: If two crossovers occur on opposing sides of an SPS, three chromosomes are linked, and one is left as a univalent. Middle panel: If two crossovers are on the same side of an SPS, as is observed more commonly in established tetraploid *A. arenosa* than in neotetraploids, the result is stable formation of two bivalents. Bias toward the desirable configuration can be achieved by increased crossover interference, allowing the crossovers at left in this example to influence the rest of both the chromosomes they link such that no additional crossovers form in the regions at right. Bottom panel: If more crossovers occur, they are generally placed such that quadrivalents form, which at least in some species can yield stable chromosome segregation and minimizes the occurrence of univalents (see text)

Autopolyploids face a distinct challenge relative to allopolyploids as they do not have differentiated sub-genomes, and generally lack recombination partner preferences. Somehow these species must sort and recombine four or more highly similar homologous chromosomes during prophase I, and come out the other end (in metaphase I) with a viable array for chromosome segregation. In most neo-autopolyploids, there are rampant problems: Multivalents (associations among three or more of the available homologs during prophase I; Fig. 3) are common and often persist to metaphase I, synapsis is often incomplete, univalents are common, laggards and bridges indicative of segregation problems are observed in anaphase I and II, and production of aneuploid gametes is common (e.g. Simonsen 1975; Weiss and Maluszynska 2000; Ramsey and Schemske 2002; Santos et al. 2003; Morgan, et al. 2021). In many neo-autotetraploids, multivalent frequency correlates negatively with fertility and can be selected against (e.g. Gilles and Randolph 1951; Bremer and Bremer-Reinders 1954; Hilpert 1957; Swaminathan and Sulbha 1959), but there is evidence, at least in grasses, that trivalent / univalent combinations are especially problematic (e.g. Myers 1945; McCollum 1957; Hazarika and Rees 1967; Crowley and Rees 1968; Simonsen 1975). For an unknown reason, problems in neopolyploid meiosis, at least in *Phlox*, seem to be worse in male than female meiosis, perhaps because female meiosis takes longer, leaving more time to correct entanglements, multivalents or univalents (Koul and Raina 1996).

As with allopolyploids, meiotic problems are rare in established autopolyploids, showing they too can evolve solutions. In most stable, established autopolyploid species, chromosomes associate primarily or exclusively as bivalents by metaphase I, but they do so without preference for particular partners, that is, they have polysomic inheritance and thus a distinct solution from that found in allopolyploids (e.g. Dawson 1941; Soltis and Rieseberg 1986; Rieseberg and Doyle 1989; Wolf et al. 1989; Qu and Hancock 1995; Hollister et al. 2012). Results from established tetraploids are consistent with the existence of genetic “multivalent suppression” systems (e.g. Hazarika and Rees 1967; Watanabe 1983), but it seems the chromosome segregation issues associated with multivalents can be solved either by increasing quadrivalent frequency at the expense of trivalent/univalent frequency (reported so far only in grasses), or increasing bivalent frequency at the expense of all types of multivalents, including trivalent/univalent combinations (common in other taxa) (Fig. 3). Despite having mostly bivalents in metaphase I, many established autopolyploids co-align all of the homologs early in prophase I and form synaptic multivalents in zygotene and pachytene, but the majority resolve before metaphase I (e.g. Hobolth 1981; Loidl 1986; Jenkins et al. 1988; Davies et al. 1990; Jones and Vincent 1994; Sybenga et al. 1994; Khazanehdari et al. 1995; Stack and Roelofs 1996; Morgan, et al. 2021). There has been speculation for many years that the dissolution of pachytene multivalents before metaphase I could involve increased crossover interference, a reduction in number of crossovers, and/or a redistribution of crossover events (e.g. Jenkins et al. 1988; Jones and Vincent 1994; Khazanehdari et al. 1995; Stack and Roelofs 1996; Bomblies et al. 2016). Detailed recent analyses have refined this view (Morgan et al. 2021). One effective way to reduce multivalents is to decrease crossing over, ideally to one event per chromosome (e.g. Watanabe 1983; Lavania 1986; Bomblies et al. 2016). Indeed, quadrivalent frequencies correlate positively with crossover frequency (Hazarika and Rees 1967), and low diploid chiasma frequency correlates with high fertility in neo-tetraploids derived from those genotypes (e.g. Hazarika and Rees 1967; Lavania 1986, 1991; Srivastava et al. 1992).

Recent work in autotetraploid *Arabidopsis arenosa* (a relative of the widely used model *A. thaliana* that exists in nature as both a diploid, and a meiotically stable autotetraploid estimated to be about 30,000 generations old (Arnold et al. 2015)) comparing diploid, neo-tetraploids and established tetraploids, provided evidence as to what both a challenge and a solution to autopolyploid meiosis can look like cytologically and molecularly. The data suggest that the meiotic stability of the tetraploid involves more than just a simple reduction in crossover number though autotetraploid *A. arenosa* does have reduced crossover number (relative to the neo-tetraploid). The established tetraploid also has shorter axis / synaptonemal complex length (Morgan et al. 2021). Across species, when DNA length is constant, synaptonemal complex length and recombination frequency are correlated (Kleckner et al. 2003). Thus, decreased axis length in the evolved tetraploids may be favored to help reduce crossover numbers (Morgan et al. 2021). In *A. arenosa* there is also a stronger bias in the established tetraploid towards crossover positioning on pachytene multivalents that yields bivalents in metaphase I (an effect mediated by crossover interference) than in the neotetraploid (Morgan et al. 2021; Fig. 3B). This stronger bias is likely accomplished through a strengthening of crossover interference, which could prevent additional crossovers from occurring elsewhere on any members of a pachytene quadrivalent that already sustained one crossover (see Fig. 3B for explanation).

Because increased crossover interference efficiency simplifies crossover patterns along single chromosomes, the *A. arenosa* solution should in principle work no matter how many chromosomes are present. Consistent with this hypothesis, when diploid *A. arenosa* was doubled twice to an octoploid state, multivalents, univalents, and asynapsis were even more rampant than in neo-tetraploids, while doubling an established tetraploid to a neo-octaploid yielded few multivalents, near complete synapsis, and little or no mis-segregation (Morgan et al. 2021). A similar pattern has also been reported in *Chrysanthemum* (Watanabe 1983). These findings suggest that adapting to polyploidy can serve as a pre-adaptation for successful meiosis at higher ploidies; the model described in *A. arenosa* suggests how this can be accomplished.

### The molecular solution(s)

*Arabidopsis arenosa:* Compared to allotetraploids, we know far less about the molecular mechanisms underlying stabilization of autotetraploid meiosis. Only *A. arenosa* has been analyzed in functional / molecular detail. Several genome scan studies of selection in the meiotically stable tetraploid lineage of *A. arenosa* identified a number of candidate genes for meiotic stabilization, which hint that this is a multigenic adaptation. Signatures of selection and differentiation were observed in genes encoding the axis proteins ASY1 and ASY3 (homologs of yeast Hop1 and Red1), the meiotic cohesin subunit REC8, the cohesin regulators PDS5, SCC2, SCC3 and SWI1, and the ZMM protein and synaptonemal complex central element ZYP1 (Hollister et al. 2012; Yant et al. 2013). All of the encoded proteins have multiple amino acid polymorphisms differentiating diploid and tetraploid alleles (Hollister et al. 2012; Yant et al. 2013; Wright et al. 2015) and most of these seem to have arisen de novo in the tetraploid lineage (Bohutinska et al. 2021a, b).

As mentioned before, we cannot speculate too confidently on untested candidates identified in genome scans for selection, but three candidate genes in *A. arenosa* have been functionally followed up. Genetic and cytological studies on plants segregating diploid versus tetraploid alleles of REC8, ASY1 and ASY3 have shown that the tetraploid alleles of these genes contribute to traits associated with meiotic stability in *A. arenosa*, such as reduced multivalent frequency (ASY1 and ASY3 only), reduced univalent frequency (REC8 only), reduced axis length (all three, but especially ASY1 and ASY3), increased crossover interference efficiency as evidenced by a stronger bias for a “good” (bivalent-favoring/non-multivalent or univalent-forming; see Fig. 3B) crossover arrangement on pachytene quadrivalents (ASY1 and ASY3) (Morgan et al. 2020, 2022). Both ASY1 and ASY3, like their yeast homologs Hop1 and Red1, are important for directing repair partner choice to homologs, and thus modulating them could affect the proportion of recombination events that mature into interhomolog vs. intersister and crossover vs. non-crossover exchanges (Schwacha and Kleckner 1997; Ferdous et al. 2012). Red1 in yeast has also been implicated in crossover interference (Zhang et al. 2014a, b, c, d), providing another possible mechanism relevant to the tetraploids, where interference efficiency increased (Morgan et al. 2021). That the structure of the axis may have changed in tetraploid *A. arenosa* is an appealing model, especially considering the possible role of the axis in providing the substrate for a chromosome tension-based model of crossover interference (Zhang et al. 2014a, b).

That the synaptonemal complex protein ZYP1 seems to have been under selection in autotetraploid *A. arenosa* (Yant et al. 2013; Bohutinska et al. 2021b) is also intriguing. In yeast and other organisms, the homologous protein Zip1 has separable critical roles in Class I crossover formation and synaptonemal complex assembly (Borner et al. 2004; de Boer and Heyting 2006; Voelkel-Meiman et al. 2015), and this dual function is also conserved in plants (Higgins et al. 2005; France et al. 2021). ZYP1 has also recently been proposed to be essential for crossover interference in *A. thaliana*, which was interpreted as meaning the synaptonemal complex mediates interference (Capilla-Perez et al. 2021; France et al. 2021). This may be, but in *A. arenosa* tetraploids, it is clear that the interference signal does not require a continuous synaptonemal complex (Morgan et al. 2021). In this regard, it is interesting that localization of MSH4 to chromosomes in yeast also depends on Zip1 (Novak et al. 2001; Shinohara et al. 2008), and in barley, ZIP1, while not strictly required for MSH4 localization, is nevertheless required for the maintenance of MSH4 foci (Barakate et al. 2014). These results link ZIP1 directly with MSH4, which in *A. thaliana* and in rice regulates crossover interference as well as crossover fate decisions prior to synaptonemal complex formation (Higgins et al. 2008; Zhang et al. 2014a, b, c, d). If the derived allele of ZYP1 in *A. arenosa* established tetraploids is important for the increased crossover interference strength in the established tetraploid, it might be this earlier role of ZIP1 in Class I interfering crossover regulation (and perhaps specifically MSH4 recruitment) that was modified by selection. Other proteins under selection also raise interesting hypotheses, for example, PDS5 is known in yeast and mice to also affect axis length and recombination patterns (Viera et al. 2020; Song et al. 2021). What roles ZYP1, PDS5 and the other genes showing evidence of selection (mostly cohesin components), remains to be tested.

*Repeatability*: As described above, the cytological solutions across different autopolyploids may be quite similar, but how repeatable the molecular basis is, is almost entirely unknown. There is now another published genome scan for selection in *Cardamine amara* (Bohutinska et al. 2021a), which is thought to be an autotetraploid (Marhold et al. 2002), and its closest diploid relative. There is little overlap between genes identified in the *C. amara* screen and those identified in *A. arenosa*, but the authors did find ploidy-differentiated amino acids in the same PDS5 paralog under selection in *A. arenosa* and in ASY3 (Bohutinska et al. 2021a). Interestingly, there is also evidence for differentiation at MSH6, which in *A. thaliana* functions together with MSH2 in mismatch recognition and repair (Culligan and Hays 2000) and plays a role in preventing ectopic recombination among non-homologous sequences (Gonzalez and Spampinato 2020). Whether MSH6 plays a role in meiotic stabilization in *C. amara* is untested, but if it does, it would hint that it may have a more “allopolyploid-like” solution. For this reason, it will be critical to test the inheritance mode of this material in addition to testing the effects of alternate alleles of candidate genes on meiosis.

### Getting over the early phase

One question that comes up frequently is how polyploid lineages ever make it through the early unstable stages that studies on neopolyploids suggest they experience. The answer is admittedly unclear. We know they *do* make it—but don’t really understand how. Two key things may contribute to early survival of polyploid lineages:

*Standing variation in diploids:* There is evidence, for example in the *B. napus* experiments described above, that standing allelic variation present in diploids can contribute to allo-polyploid stability, and that diploids with low crossover rates could be “pre-adapted” to autopolyploid meiosis (Murray et al. 1984; Srivastava et al. 1992; Jenczewski et al. 2002). In *A. arenosa*, while most of the selected polymorphisms likely arose de novo, there is evidence that an allele “part-way” to the tetraploid allele for one gene existed already in diploid *A. arenosa* (Bohutinska et al. 2021b). These results hint that some diploids may be more likely to give rise to a successful polyploids than others. When we create neopolyploids in the lab, we may be seeing a particularly dire picture—it may represent the average, but not necessarily the exact scenario that was relevant to the particular lineage that survived. Such standing variants could potentially provide neo-polyploids with enough fertility to survive long enough to allow subsequent evolutionary fine-tuning.

*Rapid meiotic stabilization of polyploids:* One of the big mysteries surrounding polyploids is something that may also be important in their early evolution—the rapid partial stabilization of meiosis observed in some systems. Though they do not generally reach the same level as a fully evolved line, meiotic stability and fertility of both neo-auto- and neo-allo-polyploids can increase noticeably after one or just a few generations, with or without direct selection for fertility and/or euploidy (e.g. Crowley and Rees 1968; Jauhar 1970; Weiss and Maluszynska 2000; Santos et al. 2003; Ferreira de Carvalho et al. 2021). Speaking against segregating standing variation in the parental gene pools being responsible for this effect, rapid partial stabilization is also observed in homozygous neotetraploid *A. thaliana* (Weiss and Maluszynska 2000; Santos et al. 2003). The rapidity of these effects (usually less than 10 or 15 generations) also largely rules out accumulation of de novo mutations. Perhaps this type of stabilization comes instead from epigenetic modifications. As with standing variation, rapid stabilization is not generally complete, but it may afford a neopolyploid a window of opportunity to overcome some of the earliest meiotic hurdles well enough to allow the slower process of genic evolution to solidify appropriate adaptations.

## Conclusions

Polyploids provide a context that can help us not only understand polyploids themselves, but also fundamental questions in meiosis, such as (i) how crossover interference is established and modified, (ii) how polymorphisms are “measured” and possibly compared among recombination partners to regulate homologous recombination maturation decisions, and (iii) how the sensitivity of mismatch recognition can be tuned (to name just a few). In terms of repeatability and predictability of the polyploid meiotic stabilization process, the molecular characterizations described above suggest that autopolyploids and allopolyploids (defined in terms of chromosome segregation behavior, not necessarily their ancestry) reliably target different aspects of meiosis that are repeatable within a type.

For allopolyploids, multivalent formation and homeologous recombination among chromosomes from different subgenomes are clearly big challenges. In established allopolyploids, this problem seems to be solved primarily by causing chromosomes within sub-genomes to preferentially recombine and segregate in meiosis I, and to avoid recombination with homeologs (what has in the literature often been referred to as a “pairing preference”, but see Box 2). So far, all known loci that cause recombination partner preferences affect either the fidelity of initial strand invasion, or the pickiness of partner choice in later stages of crossover maturation. That the molecular machines affecting these decisions are sensitive to polymorphism has long been clear, but learning how that sensitivity can be tuned is an exciting insight that might come from further mechanistically exploring this molecular feat that polyploids have accomplished.

For autopolyploids, the biggest problem seems to be the generation of multivalents among the possible homologs, particularly trivalent/univalent combinations, which have the greatest negative effect on fertility. In established autopolyploids, chromosome pairing and the earliest stages of recombination do not appear in most cases to be altered relative to the situation in Neo-polyploids, since 4-way co-alignment of axes and multivalents in pachytene are common. However, pachytene multivalents are not necess1arily maintained to metaphase I; their fate depends on crossover patterning and perhaps crossover interference, both of which have been altered in autopolyploids to bias outcomes in favor of configurations that will yield stable segregation. Whether other systems employ the same or different molecular mechanisms as *A. arenosa* will be an exciting question to explore. We also know that autopolyploid adaptation to meiosis can involve either a decrease or an increase in quadrivalent frequency, and both can help prevent univalents. Whether these distinct solutions employ related or completely different adaptations will also be interesting to explore. Another area where autopolyploids will provide important insights is in understanding how and why crossover interference strength can be modified.

There has been exciting progress in understanding the molecular basis of polyploid meiotic stabilization, but many important and interesting questions remain about how stable meiosis can be achieved when the rule that each homolog has one partner available for pairing and recombination is broken. Solving the issue of low fertility arising from meiotic problems in neo-polyploids is also an important first step for capitalizing on the high stress resilience of polyploids in novel agricultural solutions that are necessary in the face of climate change.

## References

[CR1] Alabdullah AK, Moore G, Martin AC (2021). A Duplicated copy of the meiotic gene ZIP4 preserves up to 50% pollen viability and grain number in polyploid wheat. Biology (basel).

[CR2] Allendorf FW, Danzmann RG (1997). Secondary tetrasomic segregation of MDH-B and preferential pairing of homeologues in rainbow trout. Genetics.

[CR3] Argueso JL, Wanat J, Gemici Z, Alani E (2004). Competing crossover pathways act during meiosis in Saccharomyces cerevisiae. Genetics.

[CR4] Arnold B, Kim S-T, Bomblies K (2015). Single geographic origin of a widespread autotetraploid *Arabidopsis arenosa* lineage followed by interploidy admixture. Mol Biol Evol.

[CR5] Arrigo N, Barker MS (2012). Rarely successful polyploids and their legacy in plant genomes. Curr Opin Plant Biol.

[CR6] Attia T, Röbbelen G (1986). Meiotic pairing in haploids and amphidiploids of spontaneous versus synthetic origin in rape, *Brassica napus* L. Can J Genet Cytol.

[CR7] Barakate A, Higgins JD, Vivera S, Stephens J, Perry RM, Ramsay L, Colas I, Oakey H, Waugh R, Franklin FC, Armstrong SJ, Halpin C (2014). The synaptonemal complex protein ZYP1 is required for imposition of meiotic crossovers in barley. Plant Cell.

[CR8] Benavente E, Orellana J (1989). Pairing competition between identical and homologous chromosomes in autotetraploid rye heterozygous for interstitial C-bands. Chromosoma.

[CR9] Benavente E, Orellana J (1991). Chromosome differentiation and pairing behavior of polyploids: an assessment on preferential metaphase I associations in colchicine-induced autotetraploid hybrids within the genus Secale. Genetics.

[CR10] Benavente E, Sybenga J (2004). The relation between pairing preference and chiasma frequency in tetrasomics of rye. Genome.

[CR11] Bishop DK, Zickler D (2004). Early decision; meiotic crossover interference prior to stable strand exchange and synapsis. Cell.

[CR12] Blackwell AR, Dluzewska J, Szymanska-Lejman M, Desjardins S, Tock AJ, Kbiri N, Lambing C, Lawrence EJ, Bieluszewski T, Rowan B, Higgins JD, Ziolkowski PA, Henderson IR (2020). MSH2 shapes the meiotic crossover landscape in relation to interhomolog polymorphism in Arabidopsis. EMBO J.

[CR13] Bock DG, Kane NC, Ebert DP, Rieseberg LH (2014). Genome skimming reveals the origin of the Jerusalem Artichoke tuber crop species: neither from Jerusalem nor an artichoke. New Phytol.

[CR14] Bohutinska M, Alston M, Monnahan P, Mandakova T, Bray S, Paajanen P, Kolar F, Yant L (2021). Novelty and Convergence in Adaptation to Whole Genome Duplication. Mol Biol Evol.

[CR15] Bohutinska M, Handrick V, Yant L, Schmickl R, Kolar F, Bomblies K, Paajanen P (2021). De-novo mutation and rapid protein (co-)evolution during meiotic adaptation in *Arabidopsis arenosa*. Mol Biol Evol.

[CR16] Bomblies K (2020). When everything changes at once: finding a new normal after genome duplication. Proc Biol Sci.

[CR17] Bomblies K, Madlung A (2014). Polyploidy in the Arabidopsis genus. Chromosome Res.

[CR18] Bomblies K, Higgins JD, Yant L (2015). Meiosis evolves: adaptation to external and internal environments. New Phytol.

[CR19] Bomblies K, Jones G, Franklin C, Zickler D, Kleckner N (2016). The challenge of evolving stable polyploidy: could an increase in "crossover interference distance" play a central role?. Chromosoma.

[CR20] Borner GV, Kleckner N, Hunter N (2004). Crossover/noncrossover differentiation, synaptonemal complex formation, and regulatory surveillance at the leptotene/zygotene transition of meiosis. Cell.

[CR21] Boyle WS (1950). A cytological study of Festuca Kingii. Am J Bot.

[CR22] Braz GT, Yu F, Zhao H, Deng Z, Birchler JA, Jiang J (2021). Preferential meiotic chromosome pairing among homologous chromosomes with cryptic sequence variation in tetraploid maize. New Phytol.

[CR23] Bremer G, Bremer-Reinders D (1954). Breeding of tetraploid rye in the Netherlands I. Euphytica.

[CR24] Burns R, Mandakova T, Gunis J, Soto-Jimenez LM, Liu C, Lysak MA, Novikova PY, Nordborg M (2021). Gradual evolution of allopolyploidy in Arabidopsis suecica. Nat Ecol Evol.

[CR25] Capilla-Perez L, Durand S, Hurel A, Lian Q, Chambon A, Taochy C, Solier V, Grelon M, Mercier R (2021). The synaptonemal complex imposes crossover interference and heterochiasmy in Arabidopsis. Proc Natl Acad Sci U S A.

[CR26] Chen ZJ, Ni Z (2006). Mechanisms of genomic rearrangements and gene expression changes in plant polyploids. BioEssays.

[CR27] Cifuentes M, Eber F, Lucas MO, Lode M, Chevre AM, Jenczewski E (2010). Repeated polyploidy drove different levels of crossover suppression between homoeologous chromosomes in *Brassica napus* allohaploids. Plant Cell.

[CR28] Comai L (2005). The advantages and disadvantages of being polyploid. Nat Rev Genet.

[CR29] Copenhaver GP, Housworth EA, Stahl FW (2002). Crossover interference in Arabidopsis. Genetics.

[CR30] Crickard JB, Kwon Y, Sung P, Greene EC (2019). Dynamic interactions of the homologous pairing 2 (Hop2)-meiotic nuclear divisions 1 (Mnd1) protein complex with meiotic presynaptic filaments in budding yeast. J Biol Chem.

[CR31] Crowley JG, Rees H (1968). Fertility and selection in tetraploid *Lolium*. Chromosoma.

[CR32] Culligan KM, Hays JB (2000). Arabidopsis MutS Homologs—AtMSH2, AtMSH3, AtMSH6, and a Novel AtMSH7—Form Three Distinct Protein Heterodimers with Different Specificities for Mismatched DNA. Plant Cell.

[CR33] Culligan KM, Meyer-Gauen G, Lyons-Weiler J, Hays JB (2000). Evolutionary origin, diversification and specialization of eukaryotic MutS homolog mismatch repair proteins. Nucleic Acids Res.

[CR34] Davies A, Jenkins G, Rees H (1990). Diploidization of *Lotus corniculatus* L. (Fabaceae) by elimination of multivalents. Chromosoma.

[CR35] Dawson C (1941). Tetrasomic inheritance in *Lotus corniculatus* L. J Genet.

[CR36] de Boer E, Heyting C (2006). The diverse roles of transverse filaments of synaptonemal complexes in meiosis. Chromosoma.

[CR37] De Storme N, Geelen D (2014). The impact of environmental stress on male reproductive development in plants: biological processes and molecular mechanisms. Plant Cell Environ.

[CR38] De Storme N, Copenhaver GP, Geelen D (2012). Production of diploid male gametes in Arabidopsis by cold-induced destabilization of postmeiotic radial microtubule arrays. Plant Physiol.

[CR39] Doyle J, Coate J (2019). Polyploidy, the nucleotype, and novelty: The impact of genome doubling on the biology of the cell. Int J Plant Sci.

[CR40] Emmanuel E, Yehuda E, Melamed-Bessudo C, Avivi-Ragolsky N, Levy AA (2006). The role of AtMSH2 in homologous recombination in Arabidopsis thaliana. EMBO Rep.

[CR41] Feldman M, Levy AA (2009). Genome evolution in allopolyploid wheat—a revolutionary reprogramming followed by gradual changes. J Genet Genom.

[CR42] Ferdous M, Higgins JD, Osman K, Lambing C, Roitinger E, Mechtler K, Armstrong SJ, Perry R, Pradillo M, Cunado N, Franklin FC (2012). Inter-homolog crossing-over and synapsis in Arabidopsis meiosis are dependent on the chromosome axis protein AtASY3. PLoS Genet.

[CR43] Ferreira de Carvalho J, Stoeckel S, Eber F, Lode-Taburel M, Gilet MM, Trotoux G, Morice J, Falentin C, Chevre AM, Rousseau-Gueutin M (2021). Untangling structural factors driving genome stabilization in nascent *Brassica napus* allopolyploids. New Phytol.

[CR44] Flagel LE, Wendel JF (2009). Gene duplication and evolutionary novelty in plants. New Phytol.

[CR45] France MG, Enderle Rohrig JS, Puchta H, Franklin FCH, Higgins JD (2021). ZYP1 is required for obligate cross-over formation and cross-over interference in Arabidopsis. Proc Natl Acad Sci U S A.

[CR46] Gaebelein R, Schiessl SV, Samans B, Batley J, Mason AS (2019). Inherited allelic variants and novel karyotype changes influence fertility and genome stability in *Brassica* allohexaploids. New Phytol.

[CR47] Gerton JL, DeRisi JL (2002). Mnd1p: An evolutionarily conserved protein required for meiotic recombination. Proc Natl Acad Sci.

[CR48] Gilles A, Randolph LF (1951). Reduction of quadrivalent frequency in autotetraploid maize during a period of ten years. Am J Bot.

[CR49] Gonzalez V, Spampinato CP (2020). The mismatch repair protein MSH6 regulates somatic recombination in *Arabidopsis thaliana*. DNA Repair.

[CR50] Gonzalo A, Lucas M-O, Charpentier C, Sandmann G, Lloyd A, Jenczewski E (2019). Reducing MSH4 copy number prevents meiotic crossovers between non-homologous chromosomes in Brassica napus. Nat Commun.

[CR51] Gou XY, Bian Y, Zhang A, Zhang H, Wang B, Lv R, Li J, Zhu B, Gong L, Liu B (2018). Transgenerationally precipitated meiotic chromosome instability fuels rapid karyotypic evolution and phenotypic diversity in an artificially constructed allotetraploid wheat (AADD). Mol Biol Evol.

[CR52] Grandont L, Cunado N, Coriton O, Huteau V, Eber F, Chevre AM, Grelon M, Chelysheva L, Jenczewski E (2014). Homoeologous chromosome sorting and progression of meiotic recombination in Brassica napus: ploidy does matter!. Plant Cell.

[CR53] Hazarika HM, Rees H (1967). Genotypic control of chromosome behaviour in rye. X. chromosome pairing and fertility in autotetraploids. Heredity.

[CR54] Henry JM, Camahort R, Rice DA, Florens L, Swanson SK, Washburn MP, Gerton JL (2006). Mnd1/Hop2 facilitates Dmc1-dependent interhomolog crossover formation in meiosis of budding yeast. Mol Cell Biol.

[CR55] Henry IM, Dilkes BP, Tyagi A, Gao J, Christensen B, Comai L (2014). The BOY NAMED SUE quantitative trait locus confers increased meiotic stability to an adapted natural allopolyploid of Arabidopsis. Plant Cell.

[CR56] Higgins JD, Armstrong SJ, Franklin FCH, Jones GH (2004). The *Arabidopsis MutS* homolog *AtMSH4* functions at an early step in recombination: evidence for two classes of recombination in *Arabidopsis*. Genes & Devel.

[CR57] Higgins JD, Sanchez-Moran E, Armstrong SJ, Jones GH, Franklin FC (2005). The Arabidopsis synaptonemal complex protein ZYP1 is required for chromosome synapsis and normal fidelity of crossing over. Genes Dev.

[CR58] Higgins JD, Vignard J, Mercier R, Pugh AG, Franklin FC, Jones GH (2008). AtMSH5 partners AtMSH4 in the class I meiotic crossover pathway in Arabidopsis thaliana, but is not required for synapsis. Plant J.

[CR59] Higgins EE, Howell EC, Armstrong SJ, Parkin IAP (2021). A major quantitative trait locus on chromosome A9, BnaPh1, controls homoeologous recombination in *Brassica napus*. New Phytol.

[CR60] Hilpert G (1957). Effect of selection for meiotic behaviour in autotetraploid rye. Hereditas.

[CR61] Hobolth P (1981). Chromosome pairing in allohexaploid wheat var. Chinese Spring. Transformation of multivalents into bivalents, a mechanism for exclusive bivalent formation. Carlsberg Res Commun.

[CR62] Hollister JD, Arnold BJ, Svedin E, Xue KS, Dilkes BP, Bomblies K (2012). Genetic adaptation associated with genome-doubling in autotetraploid Arabidopsis arenosa. PLoS Genet.

[CR63] Holm PB, Wang X (1988). The effect of chromosome 5B on synapsis and chiasma formation in wheat, triticum aestivum cv Chinese Spring. Carlsberg Res Commun.

[CR64] Hunter N (2015). Meiotic recombination: the essence of heredity. Cold Spring Harb Perspect Biol.

[CR65] Jakobsson M, Hagenblad J, Tavare S, Sall T, Hallden C, Lind-Hallden C, Nordborg M (2006). A unique recent origin of the allotetraploid species Arabidopsis suecica: Evidence from nuclear DNA markers. Mol Biol Evol.

[CR66] Jannoo N, Grivet L, David J, D'Hont A, Glaszmann JC (2004). Differential chromosome pairing affinities at meiosis in polyploid sugarcane revealed by molecular markers. Heredity.

[CR67] Jauhar PP (1970). Chromosome behaviour and fertility of the raw and evolved synthetic tetraploids of pearl millet, *Pennisetum typhoide*s stapf et hubb. Genetica.

[CR68] Jelenkovic G, Hough L (1970). Chromosome associations in the first meiotic division in three tetraploid clones of Vaccinium corymbosum L. Can J Genet Cytol.

[CR69] Jenczewski E, Eber F, Manzanares-Dauleux MJ, Chevre AM (2002). A strict diploid-like pairing regime is associated with tetrasomic segregation in induced autotetraploids of kale. Plant Breeding.

[CR70] Jenczewski E, Eber F, Grimaud A, Huet S, Lucas MO, Monod H, Chevre AM (2003). PrBn, a major gene controlling homeologous pairing in oilseed rape (*Brassica napus*) haploids. Genetics.

[CR71] Jenkins G, White J, Parker J (1988). Elimination of multivalents during meiotic prophase in Scilla autumnalis. II Tetraploid Genome.

[CR72] Jones GH, Vincent JE (1994). Meiosis in allopolyploid Crepis capillaris II Autotetraploids. Genome.

[CR73] Juahar PP, Riera-Lizarazu O, Dewey DG, Gill BS, Crane CF, Bennett JH (1991). Chromosome pairing relationships among the A, B, and D genomes of bread wheat. Theor Appl Genet.

[CR74] Kang HA, Shin HC, Kalantzi AS, Toseland CP, Kim HM, Gruber S, Peraro MD, Oh BH (2015). Crystal structure of Hop2-Mnd1 and mechanistic insights into its role in meiotic recombination. Nucleic Acids Res.

[CR75] Keeney S, Neale MJ (2006). Initiation of meiotic recombination by formation of DNA double-strand breaks: mechanism and regulation. Biochem Soc Trans.

[CR76] Kerzendorfer C, Vignard J, Pedrosa-Harand A, Siwiec T, Akimcheva S, Jolivet S, Sablowski R, Armstrong S, Schweizer D, Mercier R, Schlogelhofer P (2006). The Arabidopsis thaliana MND1 homologue plays a key role in meiotic homologous pairing, synapsis and recombination. J Cell Sci.

[CR77] Khazanehdari KA, Jones GH, Ford-Lloyd BV (1995). Meiosis in the leek (*Allium porrum* L.) revisited I Prophase I Pairing. Chromosome Res.

[CR78] Kleckner N, Storlazzi A, Zickler D (2003). Coordinate variation in meiotic pachytene SC length and total crossover/chiasma frequency under conditions of constant DNA length. Trends Genet.

[CR79] Koo D-H, Liu W, Friebe B, Gill BS (2017). Homoeologous recombination in the presence of Ph1 gene in wheat. Chromosoma.

[CR80] Koul KK, Raina SN (1996). Male and female meiosis in diploid and colchitetraploid Phlox drummondii Hook. (Polemoniaceae). Bot J Linn Soc.

[CR81] Krebs SL, Hancock JF (1989). Tetrasomic inheritance of isoenzyme markers in the highbush blueberry *Vaccinium corymbosum* L. Heredity.

[CR82] Lam I, Keeney S (2014). Mechanism and regulation of meiotic recombination initiation. Cold Spring Harb Perspect Biol.

[CR83] Lavania U (1986). High bivalent frequencies in artificial autopolyploids of Hyoscyamus muticus L. Can J Genet Cytol.

[CR84] Lavania U (1991). Polyploid breeding: meiosis in the diploid progenitor and its predictive value for fertility in the autotetraploid. Proc Indian National Sci Acad Part B Biol Sci.

[CR85] Le S, Griffin RA, Harwood CE, Vaillancourt RE, Harbard JL, Price A, Ngheim CQ, Koutoulis A, Nguyen KD (2021). Breeding polyploid varieties of acacia reproductive and early growth characteristics of the Allotetraploid Hybrid (*Acacia mangium × A auriculiformis*) in comparison with diploid progenitors. Forests.

[CR86] Leal-Bertioli SCM, Godoy IJ, Santos JF, Doyle JJ, Guimaraes PM, Abernathy BL, Jackson SA, Moretzsohn MC, Bertioli DJ (2018). Segmental allopolyploidy in action: Increasing diversity through polyploid hybridization and homoeologous recombination. Am J Bot.

[CR87] Li L, Jean M, Belzile F (2006). The impact of sequence divergence and DNA mismatch repair on homeologous recombination in Arabidopsis. Plant J.

[CR88] Liu Z, Adamczyk K, Manzanares-Dauleux M, Eber F, Lucas MO, Delourme R, Chèvre AM, Jenczewski E (2006). Mapping PrBn and other quantitative trait loci responsible for the control of homeologous chromosome pairing in oilseed rape (*Brassica napus* L.) haploids. Genetics.

[CR89] Loidl J (1986). Synaptonemal complex spreading in *Allium*. II. Tetraploid *A. vineale*. Can J Genet Cytol.

[CR90] Loidl J (1988). The effect of colchicine on synaptonemal complex formation in Allium ursinum. Exp Cell Res.

[CR91] Luo MC, Dubcovsky J, Dvorak J (1996). Recognition of homeology by the wheat Ph1 locus. Genetics.

[CR92] Lynn A, Soucek R, Borner GV (2007). ZMM proteins during meiosis: crossover artists at work. Chromosome Res.

[CR93] Mable BK (2004). 'Why polyploidy is rarer in animals than in plants’: myths and mechanisms. Biol J Lin Soc.

[CR94] Maherali H, Walden AE, Husband BC (2009). Genome duplication and the evolution of physiological responses to water stress. New Phytol.

[CR95] Marhold K, Huthmann M, Hurka H (2002). Evolutionary history of the polyploid complex of *Cardamine amara* (Brassicaceae): isozyme evidence. Plant Syst Evol.

[CR96] Martín AC, Alabdullah AK, Moore G (2021). A separation-of-function ZIP4 wheat mutant allows crossover between related chromosomes and is meiotically stable. Sci Rep.

[CR97] Martinez M, Naranjo T, Cuadrado C, Romero C (1996). Synaptic behaviour of the tetraploid wheat Triticum timopheevii. Theor Appl Genet.

[CR98] Martinez M, Cuñado N, Carcelén N, Romero C (2001). The Ph1 and Ph2 loci play different roles in the synaptic behaviour of hexaploid wheat *Triticum aestivum*. Theor Appl Genet.

[CR99] Mason AS, Wendel JF (2020). Homoeologous Exchanges, Segmental Allopolyploidy, and Polyploid Genome Evolution. Front Genet.

[CR100] McCollum GD (1957). Comparative studies of chromosome pairing in natural and induced tetraploid Dactylis. Chromosoma.

[CR101] Meirmans PG, Van Tienderen PH (2013). The effects of inheritance in tetraploids on genetic diversity and population divergence. Heredity.

[CR102] Mercier R, Grelon M (2008). Meiosis in plants: ten years of gene discovery. Cytogenet Genome Res.

[CR103] Mercier R, Mezard C, Jenczewski E, Macaisne N, Grelon M (2015). The molecular biology of meiosis in plants. Annu Rev Plant Biol.

[CR104] Michaelis C, Ciosk R, Nasmyth K (1997). Cohesins: Chromosomal Proteins that Prevent Premature Separation of Sister Chromatids. Cell.

[CR105] Molnar M, Bahler J, Sipiczki M, Kohli J (1995). The rec8 gene of Schizosaccharomyces pombe is involved in linear element formation, chromosome pairing and sister-chromatid cohesion during meiosis. Genetics.

[CR106] Morgan C, Zhang H, Henry CE, Franklin FCH, Bomblies K (2020). Derived alleles of two axis proteins affect meiotic traits in autotetraploid Arabidopsis arenosa. Proc Natl Acad Sci U S A.

[CR107] Morgan C, White MA, Franklin FCH, Zickler D, Kleckner N, Bomblies K (2021). Evolution of crossover interference enables stable autopolyploidy by ensuring pairwise partner connections in *Arabidopsis arenosa*. Curr Biol.

[CR108] Morgan C, Knight E, Bomblies K (2022). The meiotic cohesin subunit REC8 contributes to multigenic adaptive evolution of autopolyploid meiosis in *Arabidopsis arenosa*. PLoS Genet.

[CR109] Muramatsu M (1990). Cytogenetics of decaploid *Agropyron elongatum (Elytrigia elongata)* (2n = 70) I frequency of decavalent formation. Genome.

[CR110] Murray BG, Sieber VK, Jackson RC (1984). Further evidence for the presence of meiotic pairing control genes in Alopecurus L. (Gramineae). Genetica.

[CR111] Myers WM (1945). Meiosis in the autotetraploid Lolium perenne in relation to chromosomal behavior in autopolyploids. Bot Gaz.

[CR112] Nibau C, Gonzalo A, Evans A, Sweet-Jones W, Phillips D, Lloyd A (2022). Meiosis in allopolyploid *Arabidopsis suecica*. Plant J.

[CR113] Novak JE, Ross-Macdonald PB, Roeder GS (2001). The budding yeast Msh4 Protein Functions In Chromosome Synapsis And the regulation of crossover distribution. Genetics.

[CR114] O’Kane SL, Schaal BA, Al-Shehbaz IA (1996). The Origins of Arabidopsis suecica (Brassicaceae) as Indicated by Nuclear rDNA Sequences. Syst Bot.

[CR115] Otto SP, Whitton J (2000). Polyploid incidence and evolution. Annu Rev Genet.

[CR116] Panoli AP, Ravi M, Sebastian J, Nishal B, Reddy TV, Marimuthu MPA, Subbiah V, Vijaybhaskar V, Siddiqi I (2006). AtMND1 is required for homologous pairing during meiosis in Arabidopsis. BMC Mol Biol.

[CR117] Parisod C, Holderegger R, Brochmann C (2010). Evolutionary consequences of autopolyploidy. New Phytol.

[CR118] Parra-Nunez P, Pradillo M, Santos JL (2018). Competition for Chiasma Formation Between Identical and Homologous (But Not Identical) Chromosomes in Synthetic Autotetraploids of *Arabidopsis thaliana*. Front Plant Sci.

[CR119] Patil BD, Hardas MW, Joshi AB (1961). Auto-Alloploid Nature of *Pennisetum squamulatum* Fresen. Nature.

[CR120] Pezza RJ, Voloshin ON, Vanevski F, Camerini-Otero RD (2007). Hop2/Mnd1 acts on two critical steps in Dmc1-promoted homologous pairing. Genes Dev.

[CR121] Pikaard CS (2001). Genomic change and gene silencing in polyploids. Trends Genet.

[CR122] Pontes O, Neves N, Silva M, Lewis ML, Madlung A, Comai L, Viegas W, Pikaard CS (2004). Chromosomal locus rearrangements are a rapid response to formation of the allotetraploid Arabidopsis suecica genome. Proc Natl Acad Sci U S A.

[CR123] Premachandran MN, Prathima PT, Lekshmi M (2011). Sugarcane and polyploidy - a review. J Sugarcane Res.

[CR124] Qu L, Hancock J (1995). Nature of 2n gamete formation and mode of inheritance in interspecific hybrids of diploid Vaccinium darrowi and tetraploid *V. corymbosum*. Theor Appl Genet.

[CR125] Ramsey J, Schemske D (1998). Pathways, mechanisms, and rates of polyploid formation in flowering plants. Annu Rev Ecol Syst.

[CR126] Ramsey J, Schemske D (2002). Neopolyploidy in flowering plants. Annu Rev Ecol Syst.

[CR127] Rasmussen S (1987). Chromosome pairing in autotetraploid Bombyx males. Inhibition of multivalent correction by crossing over. Carlsberg Res Commun.

[CR128] Renny-Byfield S, Wendel JF (2014). Doubling down on genomes: polyploidy and crop plants. Am J Bot.

[CR129] Rey M-D, Martín AC, Higgins J, Swarbreck D, Uauy C, Shaw P, Moore G (2017). Exploiting the ZIP4 homologue within the wheat Ph1 locus has identified two lines exhibiting homoeologous crossover in wheat-wild relative hybrids. Mol Breeding.

[CR130] Rieseberg LH, Doyle MF (1989). Tetrasomic segregation in the naturally occurring autotetraploid Allium nevii (Alliaceae). Hereditas.

[CR131] Riley R, Chapman V (1958). Genetic Control of the Cytologically Diploid Behaviour of Hexaploid Wheat. Nature.

[CR132] Roman-Palacios C, Medina CA, Zhan SH, Barker MS (2021). Animal chromosome counts reveal a similar range of chromosome numbers but with less polyploidy in animals compared to flowering plants. J Evol Biol.

[CR133] Ross-Macdonald P, Roeder GS (1994). Mutation of a meiosis-specific MutS homolog decreases crossing over but not mismatch correction. Cell.

[CR134] Ruiz-Herrera A, Vozdova M, Fernández J, Sebestova H, Capilla L, Frohlich J, Vara C, Hernández-Marsal A, Sipek J, Robinson TJ, Rubes J (2017). Recombination correlates with synaptonemal complex length and chromatin loop size in bovids—insights into mammalian meiotic chromosomal organization. Chromosoma.

[CR135] Sakuno T, Hiraoka Y (2022). Rec8 Cohesin: A Structural Platform for Shaping the Meiotic Chromosomes. Genes.

[CR136] Salman-Minkov A, Sabath N, Mayrose I (2016). Whole-genome duplication as a key factor in crop domestication. Nature Plants.

[CR137] Salmon A, Flagel L, Ying B, Udall JA, Wendel JF (2010). Homoeologous nonreciprocal recombination in polyploid cotton. New Phytol.

[CR138] Santos JL, Orellana J, Giraldez R (1983). Pairing Competition between Identical and Homologous Chromosomes in Rye and Grasshoppers. Genetics.

[CR139] Santos JL, Alfaro D, Sanchez-Moran E, Armstrong SJ, Franklin FC, Jones GH (2003). Partial diploidization of meiosis in autotetraploid *Arabidopsis thaliana*. Genetics.

[CR140] Schwacha A, Kleckner N (1997). Interhomolog bias during meiotic recombination: meiotic functions promote a highly differentiated interhomolog-only pathway. Cell.

[CR141] Sears ER (1976). Genetic control of chromosome pairing in wheat. Annu Rev Genet.

[CR142] Serra H, Svačina R, Baumann U, Whitford R, Sutton T, Bartoš J, Sourdille P (2021). *Ph2* encodes the mismatch repair protein MSH7-3D that inhibits wheat homoeologous recombination. Nat Commun.

[CR143] Shen Y, Tang D, Wang K, Wang M, Huang J, Luo W, Luo Q, Hong L, Li M, Cheng Z (2012). ZIP4 in homologous chromosome synapsis and crossover formation in rice meiosis. J Cell Sci.

[CR144] Shinohara M, Oh SD, Hunter N, Shinohara A (2008). Crossover assurance and crossover interference are distinctly regulated by the ZMM proteins during yeast meiosis. Nat Genet.

[CR145] Simonsen O (1975). Cytogenetic investigations in diploid and autotetraploid populations of *Festuca pratensis*. Hereditas.

[CR146] Soltis DE, Rieseberg LH (1986). Autopolyploidy in Tolmiea menziesii (Saxifragaceae): genetic insights from enzyme electrophoresis. Am J Botany.

[CR147] Soltis DE, Soltis PS, Bennett MD, Leitch IJ (2003). Evolution of genome size in the angiosperms. Am J Bot.

[CR148] Soltis DE, Soltis PS, Schemske DW, Hancock JF, Thompson JN, Husband BC, Judd WS (2007). Autopolyploidy in angiosperms: have we grossly underestimated the number of species?. Taxon.

[CR149] Song K, Lu P, Tang K, Osborn TC (1995). Rapid genome change in synthetic polyploids of Brassica and its implications for polyploid evolution. Proc Natl Acad Sci.

[CR150] Song M, Zhai B, Tan T, Wang Y, Yang X, Tan Y, Chu T, Cao Y, Song Y, Wang S, Zhang L (2021). Interplay between Pds5 and Rec8 in regulating chromosome axis length and crossover frequency. Sci Adv.

[CR151] Srivastava S, Lavania UC, Sybenga J (1992). Genetic variation in meiotic behavior and fertility in tetraploid *Hyoscyamus muticus*: correlation with diploid meiosis. Heredity.

[CR152] Stack SM, Roelofs D (1996). Localized chiasmata and meiotic nodules in the tetraploid onion Allium porrum. Genome.

[CR153] Stebbins GL (1947). Types of polyploids: Their classification and significance. Adv Genet.

[CR154] Stift M, Berenos C, Kuperus P, van Tienderen PH (2008). Segregation models for disomic, tetrasomic and intermediate inheritance in tetraploids: a general procedure applied to Rorippa (yellow cress) microsatellite data. Genetics.

[CR155] Storlazzi A, Gargano S, Ruprich-Robert SG, Falque M, David M, Kleckner N, Zickler D (2010). Recombination proteins mediate meiotic spatial chromosome organization and pairing. Cell.

[CR156] Swaminathan M, Sulbha K (1959). Multivalent frequency and seed fertility in raw and evolved tetraploids of Brassica campestris var. toria. Z Vererbungsl.

[CR157] Sybenga J (1996). Chromosome pairing affinity and quadrivalent formation in polyploids: do segmental allopolyploids exist?. Genome.

[CR158] Sybenga J, Schabbink E, van Eden J, de Jong J (1994). Pachytene pairing and metaphase I configurations in a tetraploid somatic Lycopersicon esculentum× L. peruvianum hybrid. Genome.

[CR159] Szadkowski EF, Eber F, Huteau V, Lodé M, Huneau C, Belcram H, Coriton O, Manzanares-Dauleux MJ, Delourme R, King GJ, Chalhoub B, Jenczewski E, Chèvre AM (2010). The first meiosis of resynthesized Brassica napus, a genome blender. New Phytol.

[CR160] Tam SM, Hays JB, Chetelat RT (2011). Effects of suppressing the DNA mismatch repair system on homeologous recombination in tomato. Theor Appl Genet.

[CR161] Tsubouchi H, Roeder GS (2002). The Mnd1 protein forms a complex with hop2 to promote homologous chromosome pairing and meiotic double-strand break repair. Mol Cell Biol.

[CR162] Tsubouchi T, Zhao H, Roeder GS (2006). The meiosis-specific zip4 protein regulates crossover distribution by promoting synaptonemal complex formation together with zip2. Dev Cell.

[CR163] U N (1935). Genome analysis in Brassica with special reference to the experimental formation of *B. napus* and peculiar mode of fertilization. Jpn J Bot.

[CR164] Udall JA, Wendel JF (2006). Polyploidy and crop improvement. Crop Science.

[CR165] Van de Peer Y, Mizrachi E, Marchal K (2017). The evolutionary significance of polyploidy. Nat Rev Genet.

[CR166] Van Drunen WE, Husband BC (2018). Immediate vs. evolutionary consequences of polyploidy on clonal reproduction in an autopolyploid plant. Ann Bot.

[CR167] Viera A, Berenguer I, Ruiz-Torres M, Gómez R, Guajardo A, Barbero JL, Losada A, Suja JA (2020). PDS5 proteins regulate the length of axial elements and telomere integrity during male mouse meiosis. EMBO Reports.

[CR168] Vignard J, Siwiec T, Chelysheva L, Vrielynck N, Gonord F, Armstrong SJ, Schlogelhofer P, Mercier R (2007). The interplay of RecA-related proteins and the MND1-HOP2 complex during meiosis in *Arabidopsis thaliana*. PLoS Genet.

[CR169] Voelkel-Meiman K, Johnston C, Thappeta Y, Subramanian VV, Hochwagen A, MacQueen AJ (2015). Separable Crossover-Promoting and Crossover-Constraining Aspects of Zip1 Activity during Budding Yeast Meiosis. PLoS Genet.

[CR170] Watanabe K (1983). Studies on the control of diploid-like meiosis in polyploid taxa of Chrysanthemum. Theor Appl Genet.

[CR171] Watanabe Y, Nurse P (1999). Cohesin Rec8 is required for reductional chromosome segregation at meiosis. Nature.

[CR172] Weiss H, Maluszynska J (2000). Chromosomal rearrangement in autotetraploid plants of Arabidopsis thaliana. Hereditas.

[CR173] West AM, Rosenberg SC, Ur SN, Lehmer MK, Ye Q, Hagemann G, Caballero I, Uson I, MacQueen AJ, Herzog F, Corbett KD (2019). A conserved filamentous assembly underlies the structure of the meiotic chromosome axis. Elife.

[CR174] Wolf PG, Soltis PS, Soltis DE (1989). Tetrasomic inheritance and chromosome pairing behavior in the naturally occurring autotetraploid *Heuchera grossulariifolia* (Saxifragaceae). Genome.

[CR175] Wright KM, Arnold B, Xue K, Surinova M, O'Connell J, Bomblies K (2015). Selection on meiosis genes in diploid and tetraploid *Arabidopsis arenosa*. Mol Biol Evol.

[CR176] Wu SY, Culligan K, Lamers M, Hays J (2003). Dissimilar mispair-recognition spectra of Arabidopsis DNA-mismatch-repair proteins MSH2*MSH6 (MutSalpha) and MSH2*MSH7 (MutSgamma). Nucleic Acids Res.

[CR177] Wu Y, Lin F, Zhou Y, Wang J, Sun S, Wang B, Zhang Z, Li G, Lin X, Wang X, Sun Y, Dong Q, Xu C, Gong L, Wendel JF, Zhang Z, Liu B (2020). Genomic mosaicism due to homoeologous exchange generates extensive phenotypic diversity in nascent allopolyploids. National Science Review.

[CR178] Xie K-D, Xia Q-M, Wang X-P, Liang W-J, Wu X-M, Grosser JW, Guo W-W (2015). Cytogenetic and SSR-marker evidence of mixed disomic, tetrasomic, and intermediate inheritance in a citrus allotetraploid somatic hybrid between ‘Nova’ tangelo and ‘HB’ pummelo. Tree Genet Genomes.

[CR179] Xiong Y, Gan L, Hu Y, Sun W, Zhou X, Song Z, Zhang X, Li Y, Yang Z, Xu W, Zhang J, He Y, Cai D (2019). OsMND1 regulates early meiosis and improves the seed set rate in polyploid rice. Plant Growth Regul.

[CR180] Xiong Z, Gaeta RT, Edger PP, Cao Y, Zhao K, Zhang S, Pires JC (2021). Chromosome inheritance and meiotic stability in allopolyploid Brassica napus. G3 11:jkaa011. 10.1093/g3journal/jkaa011 10.1093/g3journal/jkaa011 PMC8022990 33704431

[CR181] Xu C, Bai Y, Lin X, Zhao N, Hu L, Gong Z, Wendel JF, Liu B (2014). Genome-wide disruption of gene expression in allopolyploids but not hybrids of rice subspecies. Mol Biol Evol.

[CR182] Yant L, Hollister JD, Wright KM, Arnold BJ, Higgins JD, Franklin FCH, Bomblies K (2013). Meiotic adaptation to genome duplication in *Arabidopsis arenosa*. Curr Biol.

[CR183] Zalevsky J, MacQueen AJ, Duffy JB, Kemphues KJ, Villeneuve AM (1999). Crossing over during Caenorhabditis elegans meiosis requires a conserved MutS-based pathway that is partially dispensable in budding yeast. Genetics.

[CR184] Zhang H, Bian Y, Gou X, Zhu B, Xu C, Qi B, Li N, Rustgi S, Zhou H, Han F, Jiang J, von Wettstein D, Liu B (2013). Persistent whole-chromosome aneuploidy is generally associated with nascent allohexaploid wheat. Proc Natl Acad Sci U S A.

[CR185] Zhang L, Espagne E, de Muyt A, Zickler D, Kleckner NE (2014). Interference-mediated synaptonemal complex formation with embedded crossover designation. Proc Natl Acad Sci U S A.

[CR186] Zhang L, Liang Z, Hutchinson J, Kleckner N (2014). Crossover patterning by the beam-film model: analysis and implications. PLoS Genet.

[CR187] Zhang L, Tang D, Luo Q, Chen X, Wang H, Li Y, Cheng Z (2014). Crossover formation during rice meiosis relies on interaction of OsMSH4 and OsMSH5. Genetics.

[CR188] Zhang L, Wang S, Yin S, Hong S, Kim KP, Kleckner N (2014). Topoisomerase II mediates meiotic crossover interference. Nature.

[CR189] Zhao W, Saro D, Hammel M, Kwon Y, Xu Y, Rambo RP, Williams GJ, Chi P, Lu L, Pezza RJ, Camerini-Otero RD, Tainer JA, Wang H-W, Sung P (2013). Mechanistic insights into the role of Hop2–Mnd1 in meiotic homologous DNA pairing. Nucleic Acids Res.

[CR190] Zickler D, Kleckner N (1999). Meiotic chromosomes: integrating structure and function. Annu Rev Genet.

[CR191] Zickler D, Kleckner N (2015). Recombination, pairing, and synapsis of homologs during meiosis. Cold Spring Harb Perspect Biol.

[CR192] Zickler D, Kleckner N (2016). A few of our favorite things: Pairing, the bouquet, crossover interference and evolution of meiosis. Semin Cell Dev Biol.

[CR193] Ziolkowski PA, Berchowitz LE, Lambing C, Yelina NE, Zhao X, Kelly KA, Choi K, Ziolkowska L, June V, Sanchez-Moran E, Franklin C, Copenhaver GP, Henderson IR (2015). Juxtaposition of heterozygous and homozygous regions causes reciprocal crossover remodelling via interference during Arabidopsis meiosis. Elife.

